# Reconstruction of Gut Bacteria in *Spodoptera frugiperda* Infected by *Beauveria bassiana* Affects the Survival of Host Pest

**DOI:** 10.3390/jof9090906

**Published:** 2023-09-06

**Authors:** Yuejin Peng, Shaohai Wen, Guang Wang, Xu Zhang, Teng Di, Guangzu Du, Bin Chen, Limin Zhang

**Affiliations:** Yunnan State Key Laboratory of Conservation and Utilization of Biological Resources, College of Plant Protection, Yunnan Agricultural University, Kunming 650201, China; 2021053@ynau.edu.cn (Y.P.); 15960026361@163.com (S.W.); wang_guang_17@126.com (G.W.); zhxv08@163.com (X.Z.); dtn1020@163.com (T.D.); 15288457381@163.com (G.D.)

**Keywords:** *Beauveria bassiana*, *Spodoptera frugiperda*, gut microbial diversity, virulence, *Serratia marcescens*, host defense

## Abstract

*Spodoptera frugiperda* (Lepidoptera: Noctuidae) is a migratory agricultural pest that is devastating on a global scale. *Beauveria bassiana* is a filamentous entomopathogenic fungus that has a strong pathogenic effect on Lepidoptera pests but little is known about the microbial community in the host gut and the dominant populations in fungus-infected insects. *B. bassiana* AJS91881 was isolated and identified from the infected larvae of *Spodoptera litura*. The virulence of AJS91881 to the eggs, larvae, pupae and adults of *S. frugiperda* was measured. Moreover, the gut microbial community diversity of healthy and fungus-infected insects was analyzed. Our results showed that after treatment with *B. bassiana* AJS91881, the egg hatching rate, larval survival rate and adult lifespan of the insects were significantly reduced, and the pupae rigor rate was significantly increased compared to that of the control group. Additionally, the gut microbial community was reconstructed after *B. bassiana* infection. At the phylum and genus level, the relative abundance of the Proteobacteria and *Serratia* increased significantly in the *B. bassiana* treatment group. The KEGG function prediction results showed that fungal infection affected insect gut metabolism, environmental information processing, genetic information processing, organism systems and cellular processes. Fungal infection was closely related to the metabolism of various substances in the insect gut. *Serratia marcescens* was the bacterium with the highest relative abundance after infection by *B. bassiana*; intestinal bacteria *S. marcescens* inhibited the infection of insect fungi *B. bassiana* against the *S. frugiperda*. The presence of gut bacteria also significantly reduced the virulence of the fungi against the insects when compared to the group with the larvae fed antibiotics that were infected with fungal suspension (Germfree, GF) and healthy larvae that were infected with fungal suspension prepared with an antibiotic solution (+antibiotic). In conclusion, the reconstruction of the insect intestinal bacterial community is an indispensable link for understanding the pathogenicity of *B. bassiana* against *S*. *frugiperda*. Most importantly, in the later stage of fungal infection, the increased abundance of *S. marcescens* in the insect intestine inhibited the virulence of *B. bassiana* to some extent. The findings aid in understanding changes in the gut microbiota during the early stages of entomopathogenic fungal infection of insects and the involvement of insect gut microbes in host defense mediated by pathogenic fungal infection. This study is also conducive to understanding the interaction between entomopathogenic fungi, hosts and gut microbes, and provides a new idea for the joint use of entomopathogenic fungi and gut bacteria to control pests.

## 1. Introduction

*Spodoptera frugiperda* (J.E. Smith) (Lepidoptera: Noctuidae) is a migratory agricultural Lepidoptera pest that is devastating on a global scale [1]. Its larvae are highly polyphagous with a wide host range and can feed on more than 353 species of plants, which causes huge economic losses and threats to food security [2,3,4]. *Beauveria bassiana* (Balsamo) is a filamentous entomopathogenic fungus that has a strong pathogenic effect on Lepidoptera pests [5]. When the conidia of *B. bassiana* come into contact with the cuticle of the insect, they germinate, forming bud tubes and secreting chitinase, protease and lipase to penetrate the host’s surface [6]. When the mycelium reaches the hemolymph of the insect, it quickly colonizes and infects the insect host, leading to death [7,8]. Hosts evolve new barriers in a relatively short period of time, such as antifungal peptides [9]. Pathogenic fungi, on the other hand, can overcome barriers such as the host’s external antibacterial secretions by producing enzyme families that act as ROS scavengers [10,11].

Recently, intestinal microbial communities of Lepidoptera pests have been analyzed, such as *Bombyx mori* (Linnaeus) [12], *Spodoptera littoralis* (Boisduval) [13], *Spodoptera exigua* (Hübner) [14], *Helicoverpa zea* (Boddie) [15], and *Spodoptera frugiperda* [16]. Insect gut bacteria can be broadly divided into two categories, which are important for maintaining a healthy and stable insect gut bacterial community. One category includes gut resident microbes that contribute positively to the insect’s fitness, such as promoting nutrient absorption, detoxifying harmful chemicals in food or priming the insect’s immune system [17,18]. The second category includes bacteria that are potentially harmful to the host insect [19].

However, the host can avoid death by confining these populations of pathogenic microbes to the gut [20]. Interestingly, *Serratia marcescens* Bizio is not only pathogenic to insects but it is also a symbiotic micro-organism in the insect gut [21,22]. When foreign microbes break through the early defenses, firstly, the host typically activates humoral and cellular responses to stop the infection [23,24]. Secondly, pathogenic microbes in the gut also use different strategies to overcome the host and resist the defenses [25,26]. The microbial community in the insect gut is large and the proportion of bacteria is complex and diverse. Although the gut microbiome of *S*. *frugiperda* has been elucidated, the dominant strains that play a major role in the gut have not yet been identified. More importantly, when *S*. *frugiperda* is infected by pathogenic fungi, no studies have been conducted on the changing trend of the microbial community in the host gut and the dominant populations. Additionally, insect symbionts, such as gut micro-organisms, also play important roles in host feeding, digestion, immunity, development and coevolution with insects [17]. Studies have also shown that the insect gut micro-organism plays a key role in the endocrine system and influences a variety of physiological processes, such as the supply of nutrients, the efficiency with which vector insects transmit diseases, the degradation of toxic compounds, the protection of the host against pesticides, parasites, pathogens, and the acceleration of pathogen infection and the promotion of host growth and development [27,28,29,30,31,32].

As previous studies have not focused on the gut microbial diversity of *S*. *frugiperda* infected by pathogenic fungi, we attempted to explore this aspect. This study aimed to isolate and identify the strain of *B*. *bassiana* AJS91881 and to determine the virulence of this fungus to different developmental stages of *S*. *frugiperda*. Secondly, we aimed to compare and analyze the intestinal microbial community diversity before and after fungal infection and conduct in vivo validation by eliminating the intestinal bacteria from the gut. The findings of this study could provide an important link in determining the virulence of fungi against insect hosts.

## 2. Materials and Methods

### 2.1. Strains, Media, and Insects

The *Beauveria bassiana* strain AJS91881, SHL2020, HNC-1 and DTZS19110903 strains were isolated from infected *Spodoptera litura* (Fabricius) (22°37′20.93″ N, 99°57′57.60″ E) in Lancang County, Pu’er, China in October 2019. The sabouraud dextrose agar with yeast extract medium (SDAY: 1% yeast extract, 4% glucose, 1% peptone and 1.5% agar) was cultured at a temperature of 25 °C, a photoperiod of 12L:12D and a relative humidity of 75%. After repeated purification, the pure strain was obtained and the purified strain was freeze-dried with sand and stored in the refrigerator at −80 °C for later use [33].

The coelom of the larva was cut open with a scalpel and other tissues were removed to obtain a complete worm intestine. The sample was diluted 100 times and spread on Luria-Bertani (LB: 1% tryptone, 0.5% yeast extract, 1% NaCl and 1.5% agar) plates.

The larvae were fed with fresh corn leaves for the virulence test and the adults were fed in a cage with potted corn and wet cotton (10% honey water) that was used for laying eggs. The conditions for the insect rearing were: temperature 26 °C, photoperiod 14L:10D and relative humidity 60–80% [33].

### 2.2. Molecular Identification of the Strains

After the pathogen was cultured in SDAY medium for 7 d, a Petri dish of bacteria with good growth was used to scrape off the bacteria and the cetyltrimethylammonium bromide method was used for DNA extraction [33]. The extraction followed the manufacturer’s instructions except the duration of the constant temperature water bath at 65 °C was changed from 1 h to 30 min and the TE buffer was replaced with ddH_2_O. The fungal universal primers ITSIF (5′-CTTGGTCATTTAGAGGAAGTAA-3′) and ITS4 (5′-TCCTCCGCTTATTGATATGC-3′) were used and the primers were synthesized by Shenggong BioEngineering Co., Ltd. (Shanghai, China) [34,35]. Each polymerase chain reaction (PCR) was carried out in a total volume of 25 μL, with 1 μL each of ITSIF and ITS4, 12.5 μL of PCR MasterMix, 1 μL of the DNA template and 9.5 μL of ddH_2_O. The PCR conditions were as follows: predenaturation at 95 °C for 3 min, denaturation at 94 °C for 1 min, annealing at 55 °C for 1 min and extension at 72 °C for 1.5 min for 35 cycles. Subsequently, 1% agarose gel electrophoresis was performed and the amplification results were observed using a gel imaging system to confirm amplification. Bidirectional sequencing of PCR products was performed using second-generation illumina sequencing technology. The sequencing was conducted by PCR product delivery Engineering Co., Ltd. (Shanghai, China). The sequenced ITS sequence was submitted to the NCBI website (https://blast.ncbi.nlm.nih.gov/Blast.cgi, accessed on 20 June 2023). The species and sequence items with high homology were retrieved using BLAST function. The Clustalx function of MEGA7.0 software was used to compare the sequences and the items with high homology were screened out. The phylogenetic tree was constructed by neighbor-joining [36].

### 2.3. The Virulence of Fungi against S. frugiperda

The experimental method was adopted as previously described [33]. The conidial suspension of *Beauveria bassiana* strain AJS91881 was inoculated into the eggs, 3rd instar larvae, pupae and adults using the spray method with an airbrush. Approximately 5 mL of fungal suspension was sprayed for each treatment. The fungal suspension was prepared with 0.02% Tween 80. The hatching rate of the eggs, the number of dying larvae, the number of pupae undergoing melanogenesis and the lifespan of adults were recorded daily. The concentration of the spore suspension was 10^4^, 10^5^, 10^6^, 10^7^ and 10^8^ conidia/mL. For the larvae, the fresh corn leaves were replaced every 24 h and the feces were removed. In this experiment, the number of 3rd instar larvae, pupae and adults of *S*. *frugiperda* was more than 30 per replicate and the number of eggs in each treatment was about 100. Three replicates were performed for each treatment and each experiment was repeated three times. The treatment group that was supplemented with 0.02% Tween 80 was the blank control.

### 2.4. Analysis and Identification of the Intestinal Bacterial Diversity in the Insects

In the treatment group, the 3rd instar larvae of *S*. *frugiperda* were inoculated with a 1.0 × 10^8^ conidia/mL spore suspension of the *B. bassiana* strain AJS91881(GR) and insects inoculated with 0.02% Tween 80 were used as the control (CK). On the 3rd day after inoculation, the guts of the infected and control larvae were dissected. After the larvae of the insects to be dissected were killed, the body surface was disinfected with 75% ethanol. Then, the samples were washed three times with sterile water. An insect scalpel was used to open the tail, press the tail epidermis with the blunt surface of the back of the insect scalpel, drag out the entire insect intestine and remove other tissues to obtain a complete insect intestine. Dissected insect gut samples were stored at −80 °C. Then, the insect gut tissues of the treatment and control groups were sent to Baimaik Biotechnology Co., Ltd. (Beijing, China) for Illumina NovaSeq sequencing, which can be divided into four steps: library preparation of intestinal bacterial DNA, polymerase amplification, fluorescence detection and data analysis [37]. The alpha diversity reflects the species richness and community diversity of a single sample [38,39] and it was determined using the alpha diversity index analysis software QIIME2 [40] (https://qiime2.org/) (accessed on 2 February 2022). The Chao 1 richness estimator, Shannon-Wiener diversity index and Simpson diversity index were compared [41]. Then, spatial and temporal beta diversity, a principal coordinate analysis (PCoA), was used to sort the eigenvalues and eigenvectors and select the first few eigenvalues. A PCoA can be used to find the most important coordinates in the distance matrix and the differences between individuals or groups can be observed.

The gut bacteria were isolated from the healthy 3rd instar larvae of *S*. *frugiperda* and the main bacterial species and contents between the infected and uninfected larvae were compared. The larvae of *S. frugiperda* were inoculated with four strains of *B. bassiana* (AJS91881, SHL2020, HNC-1 and DTZS19110903). On the third day after inoculation, the gut of the infected insects was selected and isolated using the LB medium plate method. According to the different dilution concentrations (10^−1^–10^−5^ times) and growth on the LB medium, the basic morphology and quantity of the bacteria were determined and molecular identification was conducted. Each plate was coated with 100 µL diluent.

### 2.5. Determination of the Influence of the Gut Micro-Organisms of S. frugiperda against B. bassiana

First, the 2nd instar larvae were fed with the drying corn leaves until the insects reached about the 3rd instar and were then soaked in a mixture of streptomycin sulfate, rifampicin, penicillin and ciprofloxacin at the same volume ratio (0.25 mg/mL) for 1 h [42]. Then, they were treated with fungal suspension. Finally, the gut was dissected and diluted into 10^1^, 10^2^, 10^3^, 10^4^ and 10^5^ solutions successively using centrifugation and coated on an LB plate to test the bactericidal effect of antibiotics.

The larvae fed antibiotics were infected with 10^8^ conidia/mL fungal suspension (Germfree, GF) and the healthy larvae were infected with 10^8^ conidia/mL fungal suspension prepared with an antibiotic solution (+antibiotic, 0.25 mg/mL), which were used to measure virulence, respectively. The 3rd instar larvae treated only with fungal spore suspension (AJS91881) was the negative control. An amount of 0.02% Tween 80 was impregnated into healthy 3rd instar larvae, which was the blank control. Each experiment was repeated three times and each treated about 30 insects. In addition, 3rd instar larvae treated with *B. bassiana* SHL2020, HNC-1 and DTZS19110903 conidia suspension of 10^8^ conidia/mL was used to determine the relative abundance of intestinal dominant bacteria.

### 2.6. Data Analysis

The observation and measurement results for all the phenotypes of all the insects and fungal strains in the three repeated experiments were analyzed using a one-way analysis of variance (ANOVA) and two-way ANOVA and the mean values among the fungal strains were compared using Tukey’s HSD test. Statistical analysis of survival data was performed by a Kaplan-Meier pairwise comparison using a log-rank test.

## 3. Results

### 3.1. Isolation and Identification of B. bassiana

**Figure 1:**
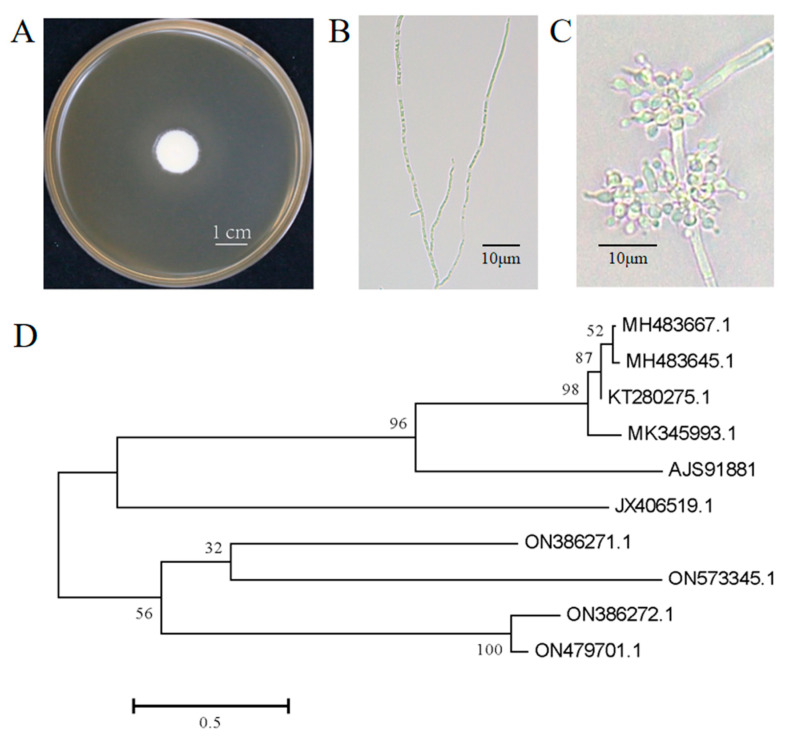
Morphological and molecular identification of *B. bassiana* AJS91881. (**A**) Colony morphology of *B. bassiana* AJS91881 on day 5 on SDAY plate. (**B**) A few slices of the five-day-old mycelium were selected and the morphology of strain AJS91881 was observed under a microscope. (**C**) The strain AJS91881 was grown for 7 days and the morphology of conidiospore and spore were observed under a microscope. (**D**) Phylogenetic analysis of strains AJS91881; relationships are depicted from neighbor joining analysis and the bootstrap values >50% from 1000 replicates are shown at the supported branch.

Strain AJS91881 was isolated from the cadaver of a diseased moth. The colony morphology of the fungus on the fifth day of growth is shown in Figure 1A and the morphology of the mycelia and conidia are shown in Figure 1B,C, respectively. The phylogenetic analysis based on the internal transcribed spacer sequence alignment showed that strain AJS91881 was closely related to *B. bassiana* and was identified as *B. bassiana* (Figure 1D).

### 3.2. The Virulence of B. bassiana to S. frugiperda

**Figure 2:**
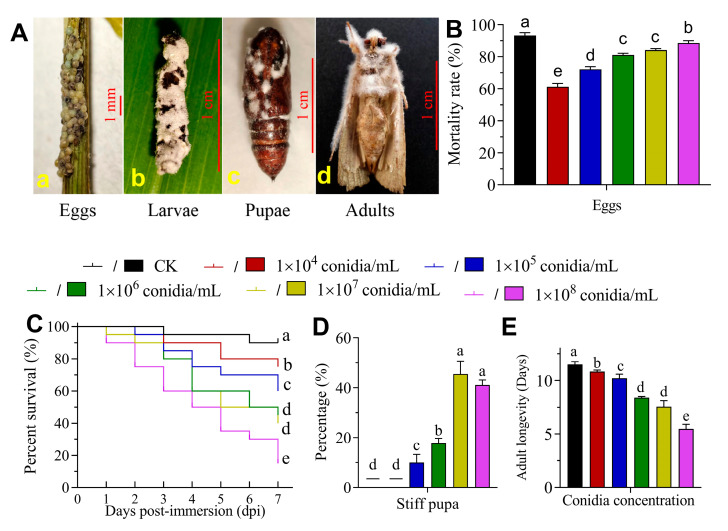
Virulence of *B. bassiana* AJS91881 to *S. frugiperda*. (**A**) Morphology of eggs (**a**), third instar larva (**b**), pupa (**c**) and adult (**d**) of *S. frugiperda* infected by *B. bassiana* AJS91881. Eggs and larvae, pupae and adults were infected by the fungus for 2, 3, 4 and 4 days, respectively. (**B**) Mortality rate of eggs of insects infected with different concentrations of fungal spore suspensions. (**C**) Survival percentage of third instar larvae infected with different concentrations of fungal spore suspensions. Statistical analysis of survival data was performed with a Kaplan-Meier pairwise comparison using a log-rank test. (**D**) Pupa rigor rate infected with different concentrations of fungal spore suspensions. Pupae were selected within 3 days of pupation. (**E**) Lifespan of adult worms infected with a suspension of fungal spores of different concentrations. Adults were selected within 3 days of emergence. a–e represented significant differences among the groups. one-way ANOVA analysis (*p* < 0.05), respectively. Tukey’s honestly significant difference [HSD]: *p* < 0.05. Error bars: standard deviation.

Figure 2A shows the symptoms of the eggs (a), third instar larvae (b), pupa (c) and adult (d) of *S. frugiperda* infected with *B. bassiana* strain AJS91881, respectively. The pathogenicity of *B. bassiana* AJS91881 against the eggs of *S. frugiperda* showed that the higher the concentration of spores, the higher the mortality rate of eggs. At 10^4^, 10^5^, 10^6^, 10^7^ and 10^8^ conidia/mL, the mortality of the eggs was 61.18 ± 2.22% (Mean ± SD), 72.16 ± 1.74%, 81.14 ± 1.07%, 84.26 ± 0.85% and 88.62 ± 1.34%, respectively (Figure 2B). The hatching rate was significantly lower than that of the control group (93.33 ± 1.73%; *p* < 0.05). The results of the virulence of strain AJS91881 to the third instar larvae of *S*. *frugiperda* showed that with the increase in the spore concentration, the cumulative mortality of the larvae gradually increased. On the seventh day of insect treatment with 10^4^, 10^5^, 10^6^, 10^7^ and 10^8^ conidia/mL, the survival rate of the third instar larvae was 75%, 60%, 45%, 40% and 15%, respectively, which were significantly lower than the 90% survival rate of the control group (*χ*^2^ = 29.77, *df* = 1, *p* < 0.001; Figure 2C). The results of the toxicity of AJS91881 to the pupae of *S*. *frugiperda* (Figure 2A–C) showed that the rate of pupae rigor increased with an increase in the concentration. The pupa rigor rate of the control group (0.02% Tween 80) and 1.0 × 10^4^ conidia/mL treatment were 0, while the pupa rigor rate of the 10^5^, 10^6^, 10^7^ and 10^8^ conidia/mL treatment was 9.99 ± 3.33%, 17.78 ± 1.92%, 45.56 ± 5.09% and 41.11 ± 1.92%, respectively (*χ*^2^ = 317.2, *df* = 5, *p* < 0.01; Figure 2D). After the adult larvae were sprayed with the *B. bassiana* AJS91881 suspension at different concentrations, the adult lifespan gradually decreased with an increase in the bacterial solution concentration and the average lifespan of the adult larvae in the control group was 11.5 ± 0.265 d. The adult lifespan of 10^4^, 10^5^, 10^6^, 10^7^ and 10^8^ conidia/mL was 10.83 ± 0.15, 10.2 ± 0.4, 8.4 ± 0.1, 7.57 ± 0.55 and 5.47 ± 0.45 d, respectively (Figure 2E). They were all significantly different from the control group (*F* = 121.3, *df* = 5, *p* < 0.05).

### 3.3. Analysis of the Abundance and Diversity of the Gut Bacteria in S. frugiperda

**Figure 3:**
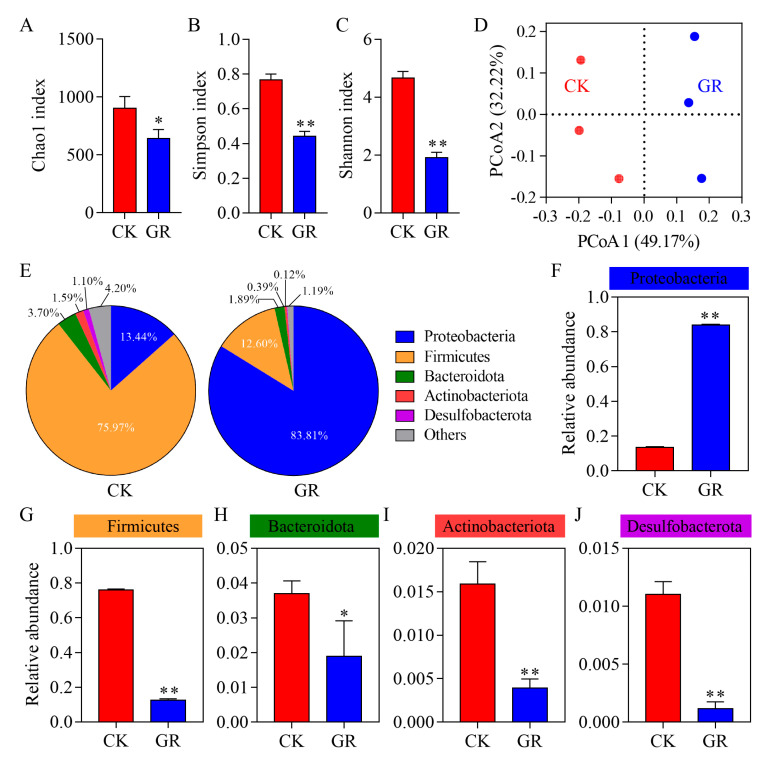
Effects of *B. bassiana* infection on gut microbial diversity of *S. frugiperda* larvae at the phylum level. (**A–C**): alpha diversity index of insect gut micro-organisms at the phylum level, including Chao1 index (**A**), Simpson index (**B**) and Shannon index (**C**). Alpha diversity refers to the species richness and evenness of a community. In the treatment group, the third instar larvae of *S*. *frugiperda* were inoculated with a 1.0 × 10^8^ conidia/mL spore suspension of the *B. bassiana* strain AJS91881(GR) and insects inoculated with 0.02% Tween 80 were used as control (CK). (**D**) Beta diversity index PCoA analysis of insect gut microbes in GR and CK groups. Beta diversity is often used to compare differences between different ecosystems, reflecting environmental heterogeneity of species. (**E**) Species distribution percentile pie chart of insect gut micro-organisms in GR and CK groups at the phylum level. (**F**–**J**) Relative abundance of gut microbes at the phylum level. Tukey’s honestly significant difference [HSD]: *p* < 0.05 (*), *p* < 0.01 (**). Error bars: standard deviation.

When compared to the control group, the Chao1 richness estimator, Shannon-Wiener diversity index and Simpson diversity index (which accounts for species richness and evenness) were significantly reduced in the larvae treated with the fungal suspension (Figure 3A–C). The beta diversity index PCoA results (Figure 3D) showed no significant overlap between the control and treatment groups based on the weighted UniFrac distance matrix, indicating that the gut microbial community structure of each cohort was differentiated. At the phylum level, the distribution of the gut bacteria species of the insects is shown in Figure 3E and the dominant bacteria in the control group were Firmicutes (75.97%) and Proteobacteria (13.44%). After being infected with *B. bassiana*, Proteobacteria was the most dominant bacteria (83.81%). The relative abundance of Firmicutes, Bacteroidota, Actinobacteria and Desulfobacterota decreased to 12.60%, 1.89%, 0.39% and 0.12%, respectively. Similarly, the results of the relative abundance difference at the phylum level showed that after *B. bassiana* infection, the relative abundance of Firmicutes, Bacteroidota, Actinobacteria and Desulfobacterota in the gut of the host had decreased significantly. Only the relative abundance of Proteobacteria increased significantly (Figure 3F–J).

### 3.4. Analysis of the Abundance and Diversity of the Gut Bacteria at the Genus Level in S. frugiperda

**Figure 4:**
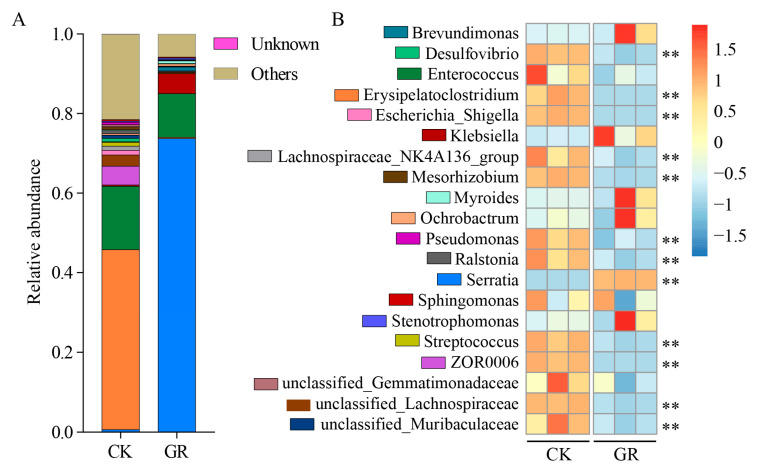
Effects of *B. bassiana* infection on gut microbes of *S. frugiperda* at the genus level. (**A,B**) Heat maps of the distribution and relative abundance of the TOP20 microbial species in the gut of fungi infected (GR) and healthy insects at the genus level. Bacterium with significant relative abundance is marked with an asterisk. Tukey’s honestly significant difference [HSD]: *p* < 0.01 (**).

The distribution of the top 20 species at the genus level showed that there were significant differences in the intestinal microbial community members between the uninfected and infected groups of *B. bassiana*. In the uninfected group, *Ochrobactrum*, ZOR006 (genus of unnamed bacteria), and *Lachnospiraceae* had a relatively high abundance, while in the *B. bassiana* infected group, the microbial community in the host gut was significantly changed. *Serratia* was significantly more abundant, accounting for more than 75% of the entire insect gut microbiome (Figure 4A). By combining the studies at the phylum and family level, heat maps were constructed using the bacterial data sets with the top 20 genera in terms of relative abundance (Figure 4B).

### 3.5. Prediction of the Microbial Function

**Figure 5:**
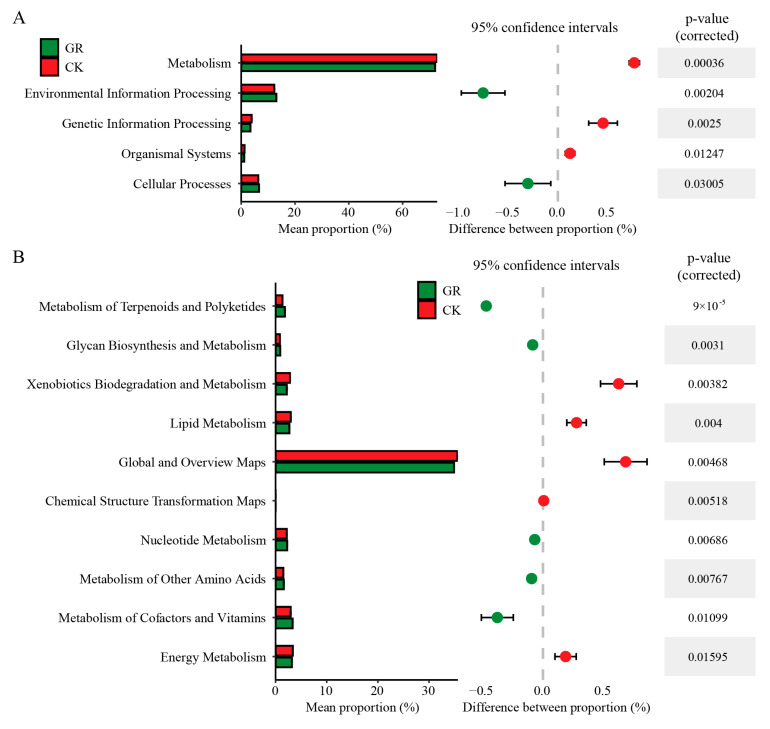
Functional prediction of gut microbes of *S. frugiperda*. (**A**) Functional prediction of host life activities in which insect gut microbes may be involved. (**B**) Functional prediction of metabolic subitems of insect gut microbes. Significant differential genes within 95% confidence intervals were selected in two groups, respectively. Tukey’s honestly significant difference [HSD]: *p* < 0.05.

To better understand the function of the gut microbiome of *S*. *frugiperda*, sequenced *16S* ribosomal RNA (rRNA) data were used to predict the function of the genes using PICRUSt2. To complete further analysis of the associations between the populations and functions, the results were compared with the Kyoto Encyclopedia of Genes and Genomes (KEGG) database (Table S1) and the top six predicted functions for which there were significant gaps between each group were assessed (Figure 5A). The fungal infections led to differences in the predicted pathway, including in metabolism, environmental information processing, genetic information processing, organismal systems and cellular processes. The abundance of functional genes in the categories of genetic information processing and cellular processes was significantly lower in the fungus-infected insect gut microbes (GR) than in the control group (CK). The abundance of functional genes in the categories of the metabolism, organismal systems and genetic information processing categories in the gut microbes of fungus-infected insects was significantly higher than that of the control group. Moreover, in the metabolic project, the metabolism of the microbial substances, including energy, cofactors and vitamins, amino acids, nucleotides and lipids, were significantly affected in the gut of the healthy and fungus-infected insects (Figure 5B; Table S2). The abundance of functional genes in intestinal micro-organisms of fungus-infected insects was significantly higher than that of the control group in the metabolism of terpenoids and polyketides, nucleotide, amino acids, cofactors and vitamins and glycan biosynthesis and metabolism. The abundance of functional genes in intestinal micro-organisms of fungus-infected insects was significantly lower than that of the control group in the xenobiotics biodegradation and metabolism, lipid metabolism, global and overview maps, chemical structure transformation maps and energy metabolism. These predicted pathways may have the most critical functions in the gut and play an important role in the overall growth and development of *S*. *frugiperda*.

### 3.6. Infection with B. bassiana Increased the S. marcescens’ Abundance in the Gut of S. frugiperda Larvae

**Figure 6:**
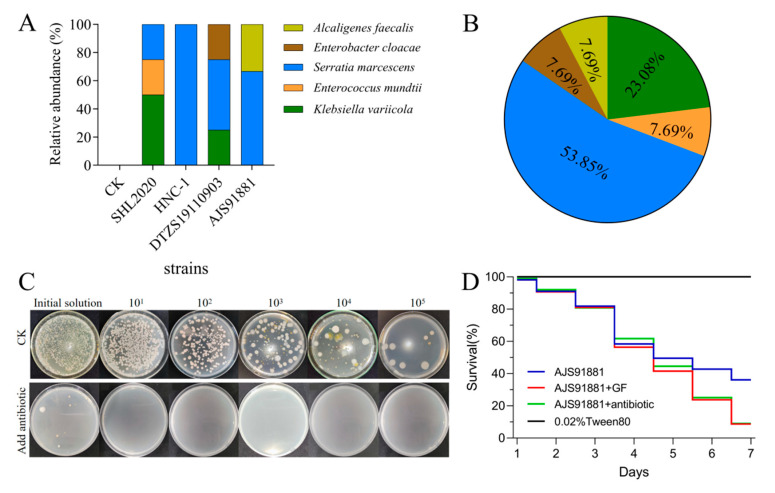
Verification of the participation of gut microbes in host resistance to *B. bassiana* infection. (**A**,**B**) The relative abundance and percentage of several strains of *B. bassiana* against the dominant bacterial species in the gut of *S. frugiperda* at the genus level. (**C**) The gut tissue homogenate cultures of third instar larvae fed with control (CK) and antibiotics (+antibiotic) were coated on LB plates. (**D**) Effect of removing gut bacteria on the third instar larvae infected with *B. bassiana* AJS91881 infection. Blank control was injected with 0.02% Tween 80 into healthy third instar larvae (black line); healthy larvae were infected with 10^8^ conidia/mL fungal suspension (blue, negative control); larvae fed antibiotics were infected with 10^8^ conidia/mL fungal suspension (red); healthy larvae were infected with 10^8^ conidia/mL fungal suspension prepared with an antibiotic solution (green).

The 16S rRNA sequencing and molecular identification of the intestinal bacteria showed that after treatment with SHL2020, HNC-1, DTZS19110903 and AJS91881, the abundance of several of the bacteria in the insect gut increased significantly, including *Alcaligenes faecalis* Castellani and Chalmers, *Enterobacter cloacae* (Jordan) Hormaeche and Edwards, *S. marcescens*, *Enterococcus mundtii* Collins and *Klebsiella variicola* Rosenblueth (Figure 6A). In the treatment group with the above four strains of *B. bassiana*, the abundant bacteria were *A. faecalis* (7.69%), *Enterobacter cloacae* (7.69%), *Serratia marcescens* (53.85%), *Enterococcus mundtii* (7.69%) and *K. variicola* (23.08%) (Figure 6B). Thus, *S. marcescens* was the bacterium with the highest relative abundance after infection by *B. bassiana*.

### 3.7. Intestinal Bacteria Influence the Virulence of Beauveria bassiana to Spodoptera frugiperda

The results of the antibiological clearance of the intestinal bacteria are shown in Figure 6C. After 3 d of culture, a few of the bacterial colonies grew in the intestinal stock solution, while no intestinal bacteria grew in the other diluted concentrations and the control group was overgrown with bacterial colonies. These results indicate that the intestinal bacteria of the third instar larvae of *S*. *frugiperda* were sensitive to the tested antibiotics and the antibiotic combination had a good effect on the removal of the intestinal bacteria of the third instar larvae of *S*. *frugiperda*. On day 4 after inoculation with *B. bassiana*, there was no significant difference in the survival rate among all the treated *S*. *frugiperda* groups. After 7 days of infection, the survival rate of *S*. *frugiperda* treated with AJS91881 was 61.7%, 90%, and 90% for the negative control, GF and add antibiotics groups, respectively (*F* = 3299, *df* = 3, *p* < 0.001, Figure 6D). The results show that the mortality rate of the third instar larvae of *S*. *frugiperda* was significantly increased after the removal of the intestinal bacteria, indicating that the larvae without intestinal bacteria were more susceptible to infection and death.

## 4. Discussion

As a representative pathogen of entomopathogenic fungi, *Beauveria bassiana* is often used to study fungal infection, the pathogenic mechanism and insect–pathogen interactions [43]. In this study, *B. bassiana* was used to infect *S. frugiperda* larvae and the healthy gut microbiome was compared to the infected gut microbiome. The results showed that the bacterial community in the host gut changed significantly at both the phylum and genus levels after infection with *B. bassiana*. Among them, the abundance of *Serratia* increased dramatically after infection and the abundance of *Yersiniaceae*, Enterobacterales and *Klebsiella* also increased. These results suggest that the mechanism of rebuilding the host gut bacterial community after infection by insect filamentous fungi is very complex, but our results do not reveal whether they determine the rebuilding of the bacterial communities.

In this study, infection with *B. bassiana* caused an increase in the abundance of *S. marcescens* in the gut of *S*. *frugiperda*, leading to a decrease in fungal virulence (Figure 6A). It has been reported that diet plays a very important role in *S. marcescens*’ transition from symbiosis to pathogenic [44]. *S. marcescens* not only acts as a pathogenic bacterium in the digestive tract of insects but also seems to participate in host defense and resistance to the infection of *B. bassiana* to some extent. As previously reported, entomopathogenic fungi that infect insects quickly colonize the hemolymph [45]. However, they do not damage the insect’s gut once they have entered the body wall, so the gut bacterial community has a chance to mount some kind of defense. Once the gut is destroyed, *Serratia* may develop outside the gut and quickly transform into a pathogenic bacterium and the insect will most likely die soon. However, although further research is needed, this is unlikely. Once the pathogenic fungus dominates the insect, its advantage becomes apparent. Thus, *S. marcescens*, which is an insect pathogen, also turns into a positive bacterium for the insect gut when attacked by entomopathogenic fungi. On the other hand, it would be difficult for bacterial communities to grow. This may be why most insects killed by pathogenic fungal infections are fossilized (fungus-lethal). In the application of biological control, on the one hand, the combination of pathogenic fungi with bacteria that can antagonize symbiotic bacteria in the gut may improve the survival rate of pests. On the other hand, we can try to force symbiotic bacteria such as *S. marcescens* to become pathogenic by combining pathogenic fungi with antibiotics or compounds that destroy insect intestinal tissue, and ultimately improve the efficiency of killing insects.

Some studies have shown that antibacterial compounds that are secreted by *Bacillus subtilis* are the main driving force of intestinal flora reconstruction [46]. For example, *S. marcescens*, which are isolated from the gut of *Nicrophorus vespilloides* produce an antimicrobial cyclic lipid peptide called serrawettin W2. The compound has strong antibacterial activity against some pathogenic bacteria [47]. Our research showed that when the four strains of *B. bassiana* infect insects, they lead to gut strains of *A. faecalis*, *Enterobacter cloacae*, *S. marcescens* and *Enterococcus mundtii*, and the relative abundance of *K. variicola* also increased significantly (Figure 6A). We hypothesize that the insect gut microbiota of *S*. *frugiperda* was reconstructed after the infection by *B. bassiana*, which is likely due to the increased abundance of the gut microbiota producing certain compounds that participate in the insect’s immune defense to some extent. In addition, host defense is essential to successfully mitigate microbial infections originating in the digestive tract [48]. Symbiotic bacteria can assist with invading pathogens [49]. For example, in bees, interfering with or depleting the gut microbiota increases the mortality of the host when attacked by the opportunistic pathogen *S. marcescens*, suggesting antagonism between *S. marcescens* and one or more members of the bee’s gut microbiota [22].

The findings suggest that fungal infection may impair the ability of *S*. *frugiperda* larvae to cope with oxidative stress, thus, negatively affecting the adaptability of the organism. Further analysis of the key metabolic pathways suggests that the gut microbiota may play different roles in fungal pathogenesis [50]. Our results also showed that infection with *B. bassiana* affected energy metabolism, amino acid metabolism and nucleic acid metabolism (Figure 5A,B).

After entomopathogenic fungi infect the body wall of insects, the ability of the fungi to grow and develop [51,52,53], withstand stress [54,55,56] and evade host defense [57] are important factors that determine the strength of their virulence. The insect gut and related micro-organisms resist the invasion and colonization of external pathogens, degrade harmful substances and produce drug resistance [58]. Infection with *B. bassiana* causes an increase in the abundance of bacteria, such as *S. marcescens*, in the insect gut. However, when the bacterial communities in the insect gut were removed by antibiotics, the virulence of *B. bassiana* was also delayed to some extent (Figure 6C,D). Many insects have symbiotic microbiota in their gut, which provide the host insect with evolutionary advantages, including protecting the host from antagonists and degrading toxins [59]. Therefore, gut bacteria may play a temporary role in protecting insects from pathogenic bacteria, which is an important part of determining the pathogenicity of *B*. *bassiana* against *S*. *frugiperda*. Notably, in the assay for fungal action with or without antibiotics on the larvae, the effect of the fungal infection on the larvae fed with antibiotics was similar to what was observed in the larvae inoculated by an antibiotic-inclusive fungal suspension (Figure 6C,D). This suggests that both methods of antibiotic use have inhibitory effects on insect gut bacteria.

The results of this study will provide new insights into the factors that determine the virulence of pathogenic fungi and the involvement of host gut microbes against infection by pathogenic fungi. However, our work fails to explain whether insect gut microbes cause differences in the virulence of pathogenic fungi, which is further explored.

## 5. Conclusions

The results of this study showed that the intestinal microbial community was reconstructed after *Beauveria bassiana* infection, which significantly changed in the relative abundance of bacteria both at the phylum level and at the genus level. The KEGG functional analysis showed that fungal infection had important effects on the gut metabolism and life activities of insects. *Serratia marcescens* was the bacterium with the highest relative abundance after infection by *B. bassiana*. The presence of gut bacteria also affects the virulence of the fungus against the insects. Overall, most bacteria were killed by antibiotics after the antibiotic treatment, so the difference in virulence could be related to an unprotected gut. The involvement of the insect gut bacteria in host defense provides further understanding in determining the pathogenicity of *B. bassiana* against *Spodoptera frugiperda*. Our results also help to understand the interaction between entomopathogenic fungi, host and intestinal microbes and provide a new idea for the joint control of insect pests by entomopathogenic fungi and gut bacteria.

## Data Availability

The data that support the findings of this study are openly available in at https://www.mdpi.com/ethics.

## Supplementary Materials

The following supporting information can be downloaded at: https://www.mdpi.com/article/10.3390/jof9090906/s1, Table S1: Prediction of KEGG function on gut microbial community of *Spodoptera frugiperda*; Table S2: KEGG function prediction on metabolic items of gut microbial community of *Spodoptera frugiperda*.
