# Supplementary material for: Reconstruction of Gut Bacteria in Spodoptera frugiperda Infected by Beauveria bassiana Affects the Survival of Host Pest
Source: J Fungi (Basel). 2023 Sep 6;9(9):906. doi: 10.3390/jof9090906 (PMC10532432; doi:10.3390/jof9090906)
Supplement: Supplementary file 1 [file jof-09-00906-s001.zip › jof-2546480-supplementary.pdf]

Table S1. Prediction of KEGG function on gut microbial community of *Spodoptera frugiperda.*

class1	GR: mean rel.freq.(%)	GR: std.dev.(%)	CK: mean rel.freq.(%)	CK: std.dev.(%)	p-values	p-values (corrected)	Difference between means	95.0% lower CI	95.0% upper CI
Cellular Processes	6.644629724	0.002059388	6.341108575	0.076240477	0.030051251	0.030051251	0.30352115	0.071803566	0.535238733
Environmental Information Processing	13.06198998	0.078929925	12.30513833	0.080180896	0.00068238	0.002047139	0.756851647	0.535942051	0.977761243
Genetic Information Processing	3.453069432	0.057760081	3.91133006	0.041499132	0.001251029	0.002502059	-0.458260629	-0.603680685	-0.312840572
Human Diseases	3.676107046	0.011997437	3.379759588	0.05605883	0.01425082	0.017100984	0.296347458	0.135207762	0.457487154
Metabolism	72.03731185	0.007524956	72.8121218	0.019823997	6.11E-05	0.000366696	-0.774809955	-0.827452826	-0.722167084
Organismal Systems	1.12689197	0.016593985	1.250541641	0.001770213	0.008314658	0.012471987	-0.123649672	-0.173354846	-0.073944497

Table S2. KEGG function prediction on metabolic items of gut microbial community of *Spodoptera frugiperda*.

class2	GR: mean rel.freq.(%)	GR: std.dev.(%)	CK: mean rel.freq.(%)	CK: std.dev.(%)	p-values	Pvalues	Difference between means	95.0% lower CI	95.0% upper CI
Metabolism of Terpenoids and Polyketides	1.823884182	0.006678268	1.35657946	0.009545711	2.03205E-06	0.00009	-0.467304722	-0.443333352	-0.491276092
Glycan Biosynthesis and Metabolism	0.924920699	0.004449329	0.840449885	0.000690466	0.001102417	0.00310	-0.084470814	-0.071357567	-0.097584062
Xenobiotics Biodegradation and Metabolism	2.195608517	0.055944703	2.8206467	0.020332046	0.001614038	0.00382	0.625038183	0.7746766	0.475399766
Lipid Metabolism	2.699200496	0.016558886	2.976679027	0.031774025	0.001601112	0.00400	0.277478531	0.357930185	0.197026877
Global and Overview Maps	34.89326025	0.021194618	35.57553522	0.064833165	0.002183196	0.00468	0.68227497	0.858688583	0.505861357
Chemical Structure Transformation Maps	0.002266068	0.000653023	0.009212645	0.000176348	0.002645388	0.00518	0.006946577	0.008773789	0.005119365
Nucleotide Metabolism	2.262022594	0.011160344	2.194589425	0.007013484	0.003659788	0.00686	-0.06743317	-0.039517237	-0.095349102
Metabolism of Other Amino Acids	1.614876987	0.0043486	1.521908393	0.012184517	0.004260114	0.00767	-0.092968594	-0.060280307	-0.125656882
Metabolism of Cofactors and Vitamins	3.340825152	0.000648359	2.964843724	0.043418496	0.006592405	0.01099	-0.375981427	-0.243925172	-0.508037683
Energy Metabolism	3.197674597	0.005578466	3.384577623	0.030021112	0.010630675	0.01595	0.186903026	0.274289422	0.099516631
